# Author Correction: A RAB35-p85/PI3K axis controls oscillatory apical protrusions required for efficient chemotactic migration

**DOI:** 10.1038/s41467-018-04515-y

**Published:** 2018-05-22

**Authors:** Salvatore Corallino, Chiara Malinverno, Beate Neumann, Christian Tischer, Andrea Palamidessi, Emanuela Frittoli, Magdalini Panagiotakopoulou, Andrea Disanza, Gema Malet-Engra, Paulina Nastaly, Camilla Galli, Chiara Luise, Giovanni Bertalot, Salvatore Pece, Pier Paolo Di Fiore, Nils Gauthier, Aldo Ferrari, Paolo Maiuri, Giorgio Scita

**Affiliations:** 1 IFOM, the FIRC Institute of Molecular Oncology, Via Adamello 16, 20139 Milan, Italy; 2 Advanced Light Microscopy Facility, European Molecular Biology Laboratory, Meyerhofstr. 1, 69117 Heidelberg, Germany; 3 ETH Zurich, Laboratory of Thermodynamics in Emerging Technologies, Sonneggstrasse 3, 8092 Zurich, Switzerland; 4 Department of Experimental Oncology, European Institute of Oncology, via Adamello 16, 20139 Milan, Italy; 5 Department of Oncology and Hemato-Oncology, University of Milan, Via Festa del Perdono 7, 20122 Milan, Italy

Correction to: *Nature Communications* 10.1038/s41467-018-03571-8, published online 16 April 2018

The originally published version of this Article contained an error in the name of the author Salvatore Corallino, which was incorrectly given as Corallino Salvatore. This has now been corrected in both the PDF and HTML versions of the Article.

