# Supplementary material for: A RAB35-p85/PI3K axis controls oscillatory apical protrusions required for efficient chemotactic migration
Source: Nat Commun. 2018 Apr 16;9:1475. doi: 10.1038/s41467-018-03571-8 (PMC5902610; doi:10.1038/s41467-018-03571-8)
Supplement: Supplementary file 2 — Description of Additional Supplementary Files [file 41467_2018_3571_MOESM2_ESM.pdf]

Description of Supplementary Files

File Name: Supplementary Movie 1
Description: **CDRs are sites of macropinocytic internalization** MEF cells were serum starved for 2 h and stimulated with PDGF (20 ng/ml). CDR formation was monitored by time-lapse microscopy. Pictures were acquired by differential interference contrast (DIC) imaging every 20 sec over a period of 40 min The white box highlights the macropinosomes formation. Scale-bar, 50µm.

File Name: Supplementary Movie 2
Description: **RAB35 upregulation is sufficient to promote multiple CDR formation** MEF Ctrl, RAB35-expressing and RAB35-silenced cells were cultured in complete media and monitored by time-lapse phase contrast microscopy in order to score CDR formation. Pictures were taken every 30 sec over a 1 h period. Scale-bar, 50µm.

File Name: Supplementary Movie 3
Description: **CDR formation in response to local delivery of PDGF** MEF cells were cultured in absence of serum for 1 h. The green arrow indicates the point of PDGF (20 ng/ml) local delivery. CDR formation and subsequent protrusion extension was recorded by time-lapse phase contrast microscopy every 20 sec over a period of 20 min. Scale-bar, 20µm.

File Name: Supplementary Movie 4
Description: **RAB35-dependent CDR formation precedes the extension of protrusions at the leading edge.** MEF pSLIK HA-RAB35 GFP-LifeAct cells were cultured in presence or absence of doxycycline to induce RAB35 expression (+DOX). The dynamics of CDR formation and protrusion extension was monitored by time-lapse fluorescence microscopy every 30 sec over 1 h period. Scale-bar, 50µm.

File Name: Supplementary Movie 5
Description: **CDR travels along and in synchrony with the extended protrusions** MEF pSLIK HA-RAB35 GFP-LifeAct cells were cultured in presence or absence of doxycycline to induce RAB35 expression (+DOX). Live cells were imaged by time-lapse fluorescence microscopy to monitor travelling CDRs. Images were taken every 30 sec over 21 min period. Scale-bar, 20µm.

File Name: Supplementary Movie 6
Description: **RAB35 promotes recurrent waves of CDR formation** MEF pSLIK HA-RAB35 GFP-LifeAct cells were cultured in presence of doxycycline to induce RAB35 expression. The frequency of CDR formation in the same region was captured by timelapse fluorescence microscopy. GFP-LifeAct images were acquired every 30 sec over 1 h period. Scale-bar, 10µm.

File Name: Supplementary Movie 7
Description: **RAB35 downregulation affects the response to chemotactic gradients** MEF Ctrl (shCtrl) and RAB35-silenced (shRAB35) cells were loaded into IBIDI chemotactic chambers and subjected to a PDGF chemotactic gradient (0 – 20 ng/ml). Cell locomotion was followed by time-lapse phase contrast microscopy for a 24 h period taking pictures every 5 min. Scale-bar, 100µm.

File Name: Supplementary Movie 8
Description: **Random cell motion upon perturbation of RAB35 levels** MEF Ctrl (shCtrl), RAB35-silenced (shRAB35) and RAB35- expressing (HA-RAB35) cells were cultured in complete media and the locomotion was monitored by time-lapse phase contrast microscopy. Pictures were taken every 5 min over a 24 h period. Scale-bar, 100µm.

File Name: Supplementary Movie 9
Description: **RAB35 silencing has no effect kenotaxis in cells migrating to close wound** Scrambled and *Rab35*-silenced (siRab35) MEFs seeded to form a confluent monolayer, were scratched with a pipette tip and time lapse recording started immediately after. Images were taken every 5 min over 24 h. Scale Bar, 50 µm.

File Name: Supplementary Movie 10
Description: **Lack of RAB35 impairs cell locomotion on suspended Fibronectin-coated fibers.** MEF Ctrl (shCtrl) and RAB35-silenced (shRAB35) cells were seeded on suspended nanofibers coated with Fibronectin. Cell migration was followed by time-lapse phase contrast microscopy for about 6 h. Images were taken every 1 min.

File Name: Supplementary Movie 11
Description: **Lack of RAB35 impairs cell locomotion on microprinted Fibronectin-lines** MEF Ctrl (shCtrl) and RAB35-silenced (shRAB35) cells were seeded on Fibronectin-coated lines of 10 µm width and imaged by time-lapse phase contrast microscopy every 5 min for 10 h. Scale-bar, 100µm.

File Name: Supplementary Movie 12
Description: **RAB35 ablation in MCF10.DCIS.com cells reduces chemoinvasion into 3D matrices of native type I collagen** The capability of MCF10.DCIS.com shRNA Ctrl and shRNA RAB35 to penetrate and invade collagen type I gels was monitored by time-lapse phase contrast microscopy. Images were acquired every 10 min over a 48 h period. Scale-bar, 100µm.

File Name: Supplementary Movie 13
Description: **RAB35-induced CDRs are RAB5- and RAC-dependent but PDGFRB-independent** MEF pSLIK HA-RAB35 cells were interfered for PDGFRB, RAB5a,b,c and RAC and cultured in presence or absence of doxycycline to induce RAB35 expression (+DOX). Scrambled cells were used as control. The dynamics of CDR formation was monitored by time-lapse phase contrast microscopy every 30 sec over 1 h period. Scale-bar, 100µm.

File Name: Supplementary Movie 14
Description: **Chemical inhibition of PI3K abrogates RAB35-dependent dorsal ruffles formation** MEF pSLIK HA-RAB35 cells were cultured in presence or absence of doxycycline to induce RAB35 expression (+DOX). 2 h before live imaging session, cells were treated (LY294002) or not (Ctrl) with the PI3K chemical inhibitor. Samples were imaged every 30 sec for 1 h by time-lapse phase contrast microscopy. Scale-bar, 50µm.

File Name: Supplementary Movie 15
Description: **Genetic ablation of the regulatory subunit of PI3K affects the RAB35-dependent CDR formation** MEF p85 -/- pSLIK HA-RAB35 cells were cultured in presence or absence of doxycycline to induce RAB35 expression (+DOX). Samples were imaged by time-lapse phase contrast microscopy every 30 sec for 1 h period. Scale-bar, 50µm.

File Name: Supplementary Movie 16
Description: **Tumor-associated, hyperactive mutants of RAB35 promote constitutive CDR formation** MEFs infected with pSLIK-HA-RAB35, or –HA-RAB35A151T or -HA-RAB35F161L were incubated either in the absence (-Dox) or presence (+Dox) of Doxycycline and monitored for 1 hour by time-lapse phase contrast microscopy in the absence of any added PDGF. Samples were imaged every 30 sec for 1h period. Scale-bar, 50µm.

File Name: Supplementary Data 1
Description: **List and position within each 96-wells plate of the siRNA used in the screening.** For each siRNA (siRNA ID) used, we report the plate (Plate name) in which it is spotted, its position within the plate (Row and Column), the Gene symbol, the oligo sequences and RefSeq ID of the corresponding gene. The oligo arrays were purchased from AMBION.

File Name: Supplementary Data 2
Description: **CDR-score for each of the siRNA oligo tested.** CDR-score for each of the siRNA oligo tested across the various 96-wells plates. The relative CDR score with respect to control (siEGFP—siEGFP) for each siRNA against the various RABs is reported (see methods for details). The number of plate, images and cells analyzed for each siRNA is indicated.
