# Supplementary material for: A RAB35-p85/PI3K axis controls oscillatory apical protrusions required for efficient chemotactic migration
Source: Nat Commun. 2018 Apr 16;9:1475. doi: 10.1038/s41467-018-03571-8 (PMC5902610; doi:10.1038/s41467-018-03571-8)
Supplement: Supplementary file 3 — Supplementary Information [file 41467_2018_3571_MOESM3_ESM.pdf]

Supplementary Information

A RAB35-p85/PI3K AXIS CONTROLS OSCILLATORY APICAL PROTRUSIONS REQUIRED FOR EFFICIENT CHEMOTACTIC MIGRATION

By Corallino et al.

1. Supplementary Figure Legends

1. Supplementary Figure Legends

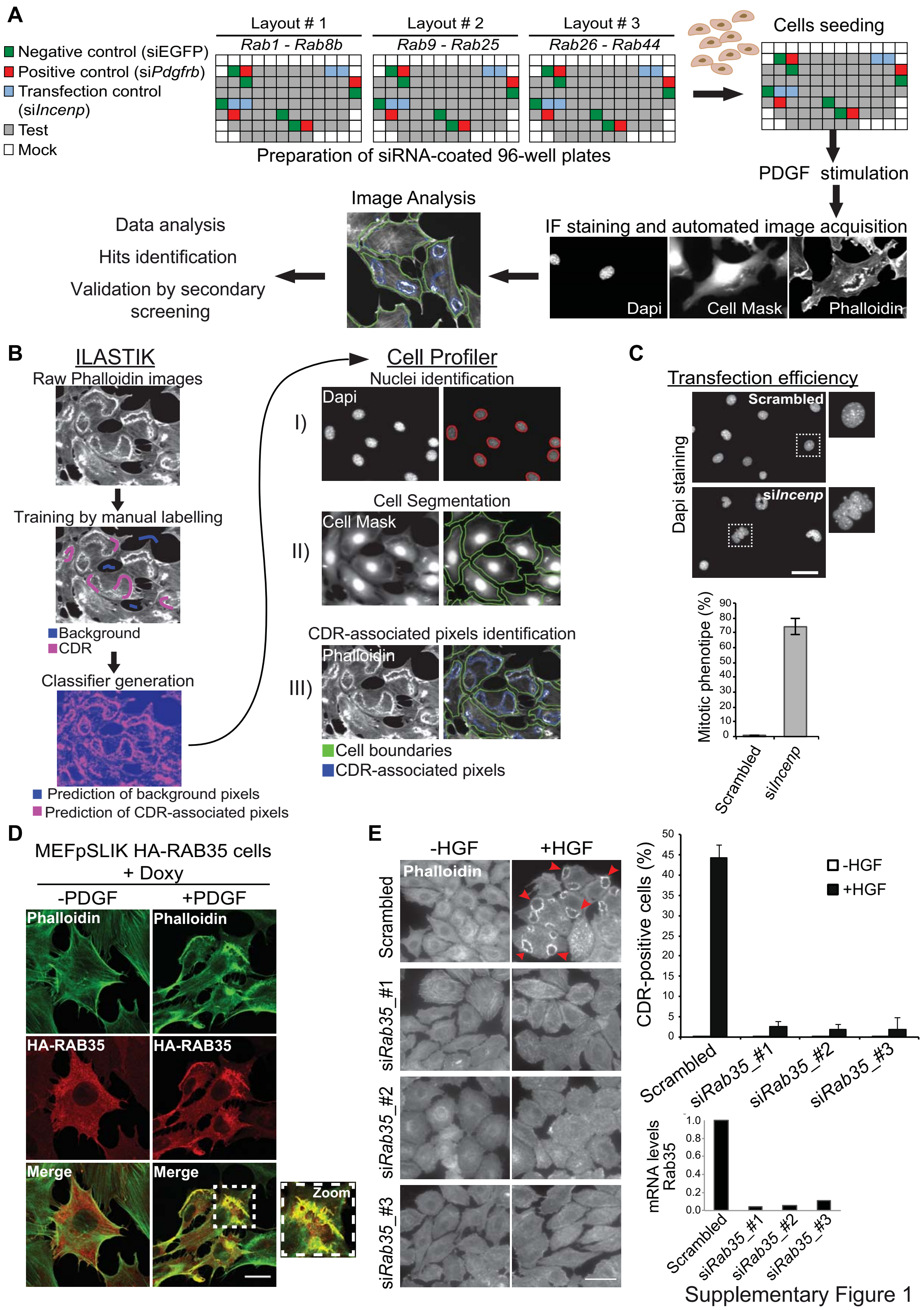

Supplementary Figure 1

Supplementary Figure 1. Outline of the screening pipeline and results

(A) Scheme of the RNAi-based screening. Glass-bottom 96-well plates were coated with siRNAs targeting RAB GTPase family members and transfection reagent. After 48h, cells were serum starved for 2h and stimulated with PDGF (20 ng/ml) for 10’. Cells were subjected to IF staining to

detect cell nuclei (Dapi), Membrane (Cell Mask) and F-actin (Phalloidin). Images were automatically acquired by an Olympus ScanR microscope and processed through a dedicated-image analysis pipeline to quantify CDR-associated pixels and to generate a list of CDR-regulators.

(B) Image analysis pipeline. A subset of randomly chosen images acquired in the GFP channel (Phalloidin) were used to manually define CDR-associated pixels and build a Random Forest classifier by using the ilastik software (http://ilastik.org/). The generated classifier was combined to a dedicated Cell Profiler (http://www.cellprofiler.org/) pipeline. Dapi staining was used to identify nuclei and Cell Mask to perform cell segmentation. Following the application of a number of filters, CDR-associated pixels were identified in the FITC-Phalloidin stained images.

(C) Dapi staining of scrambled and *Incenp*-depleted MEFs. The percentage of morphological altered nuclei is expressed as mean ± SD (n > 300 cells/condition in 3 independent experiments). Scale-bar, 50 μm. Boxes indicate magnified areas shown on the right.

(D) Serum-starved, doxycycline-treated pSLIK-HA-RAB35-human-MEFs were stimulated with PDGF for 10’ or mock treated, fixed and co-stained with FITC-Phalloidin and anti-HA antibody to detect F-actin and RAB35, respectively. Representative fluorescence images are reported for each experimental condition. The box indicates magnified area shown on the right. Scale-bar, 50 μm.

(E) RAB35 downregulation impairs CDRs formation in HGF-stimulated HeLa cells. *Left*: HeLa cells were interfered for RAB35 using 3 different oligos. A scrambled oligo was used as negative control. After 48 h, cells were serum starved for 6h and stimulated with HGF or mock treated for 10’. Samples were stained with Phalloidin. Representative images are reported for each experimental condition. Arrows indicate CDRs. Scale-bar, 50μm. *Right*: CDRs were manually counted and the percentage of CDR-positive cells is reported. Data are the mean ± SD (n > 200 cells/condition in three independent experiments). Target gene silencing was verified by qRTPCR.

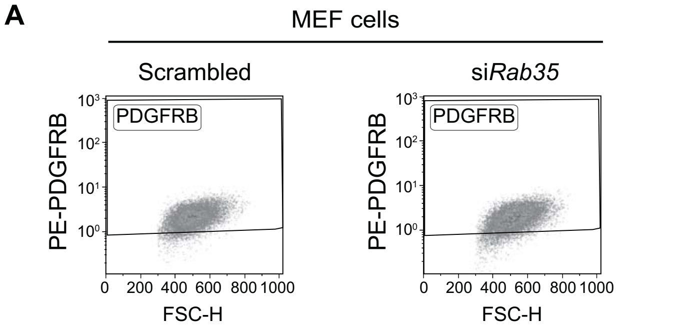

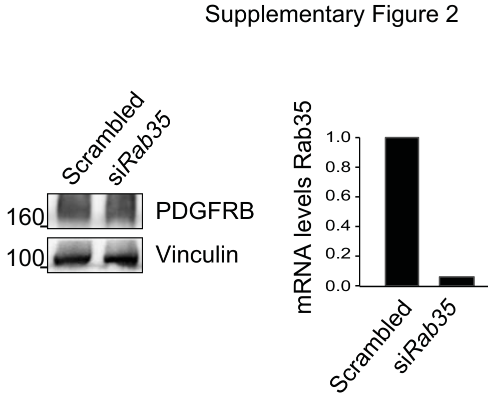

Gate	Number	%Total	%Gated
All	19,855	96.8962	100.0000
PDGFRB	19,114	93.2800	96.2679

Gate	Y-Med	Y-GMean
All	2.0283	1.9932
PDGFRB	2.0647	2.0740

Gate	Number	%Total	%Gated
All	19,886	96.2443	100.0000
PDGFRB	18,448	89.2847	92.7688

Gate	Y-Med	Y-GMean
All	1.9397	1.8424
PDGFRB	2.0264	2.0365

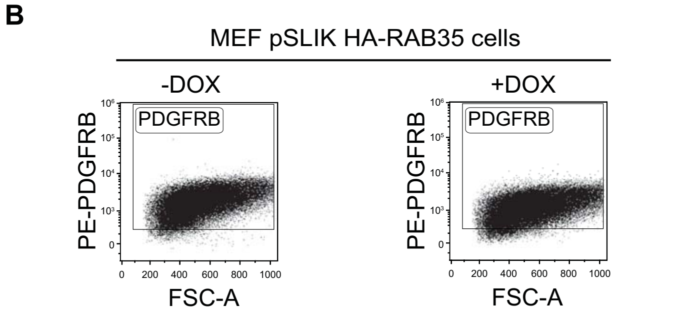

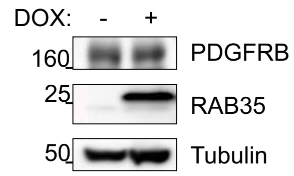

Gate	Number	%Total	%Gated
All	54,771	86.9478	100.0000
PDGFRB	54,098	85.8794	98.7712

Gate	Y-Med	Y-GMean
All	1,493.7439	1,689.2534
PDGFRB	1,508.4730	1,435.0806

Gate	Number	%Total	%Gated
All	56,621	81.2295	100.0000
PDGFRB	55,380	79.4491	97.8082

Gate	Y-Med	Y-GMean
All	1,187.7831	1,326.8330
PDGFRB	1,207.4240	1,156.6849

Supplementary Figure 2. RAB35 does not alter total and cell surface level of PDGFRB

(A) Cell surface and total levels of PDGFRB are not affected by *Rab35* silencing. MEFs were interfered for *Rab35*. Plasma-membrane (*left*) and total (*middle*) levels of PDGFRB were measured by FACS and Western blot with the indicated abs, respectively. The silencing of the target gene was verified by qRTPCR (*right*).

(B) Cell surface and total levels of PDGFRB are not affected by RAB35 ectopic expression. Surface (*left*) and total (*right*) levels of PDGFRB in doxycycline-treated pSLIK-HA-RAB35 (human)-MEFs were measured by FACS and Western blot with the indicated antibodies, respectively.

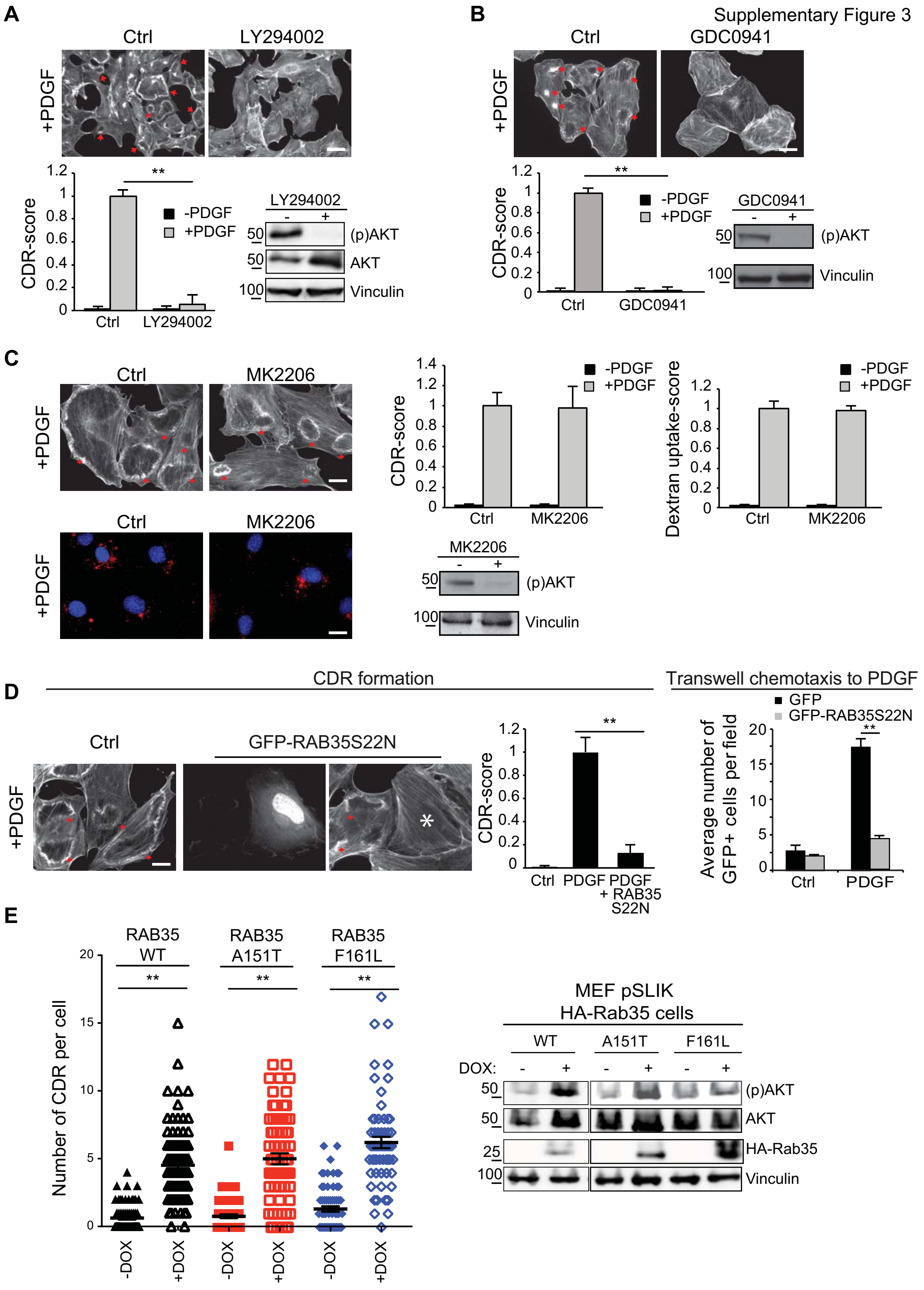

Supplementary Figure 3. Impact on CDRs of perturbing PI3K, AKT and GTP/GDP-RAB35 cycles

(A-B) MEFs pre-incubated with vehicle or PI3K inhibitors, LY294002 or GDC0941 were stimulated or mock-treated with PDGF. CDR-score was calculated by normalizing the number of CDR-positive cells per each condition against vehicle-treated, PDGF-stimulated samples. Scale-bar, 30 µm. Data are mean ± SD (n = 150 cells/condition, 2 independent experiments). **$p < 0.01$,

paired Student's t-test. AKT phosphorylation was assessed by immunoblotting. Red arrows indicate CDRs.

(C) MEFs pre-incubated with vehicle or MK-2206 (AKT inhibitor) were stimulated with PDGF for 10' to measure CDRs (upper leftmost panels, red arrows indicate CDRs) or for 30' in the presence of tetramethyl-rhodamine-dextran (lower leftmost panels) to monitor micropinocytosis. Scale-bar, 10 µm. CDR-score was calculated as described above (middle upper graph). Dextran uptake quantified as total cell fluorescence/cell (rightmost graph) is expressed as A.U. after normalizing to control PDGF-treated cells. Data are the mean ± SD (n= 750 cells/condition in 3 independent experiments). AKT phosphorylation and Vinculin levels were assessed by immunoblotting.

(D) Serum-starved MEFs transfected with an empty or a GFP-RAB35S32N vector were stimulated with PDGF for 10', and stained with phalloidin or processed for epifluorescence. Red arrows indicate CDRs. Asterisk indicates RAB35S22N-expressing cells. The CDR-score of RAB35S22N-expressing and control cells was as in (C). Scale-bar, 10 µm. Data are mean ± SD (n = 50 cells/condition, 3 independent experiments). $^{**}p < 0.01$, paired Student's t-test. Leftmost graph, migration toward PDGF (20 ng/ml) of GFP (Ctrl) and GFP-*RAB35*S22N-expressing MEFs were assessed in Transwell assays as described in Figure S2D and quantified as average number of GFP-expressing cells per field ± SD (at least 8 fields of view/condition, 2 independent experiments). Data are the mean ± SD, ** $p < 0.01$.

(E) MEFs infected with pSLIK-HA-RAB35, or –HA-RAB35A151T or -HA-RAB35F161L were incubated either in the absence (-Dox) or presence (+Dox) of doxycycline and monitored for 1h by time-lapse (Movie 16) in the absence of PDGF. The number of CDRs/cell formed in 1h is expressed as mean ± SEM (n = 80 cells/condition, 3 independent experiments). $^{**}p < 0.01$, paired Student's t-test. RAB35, phosphor and total AKT Vinculin and levels were determined by immunoblotting.

Supplementary Figure 4

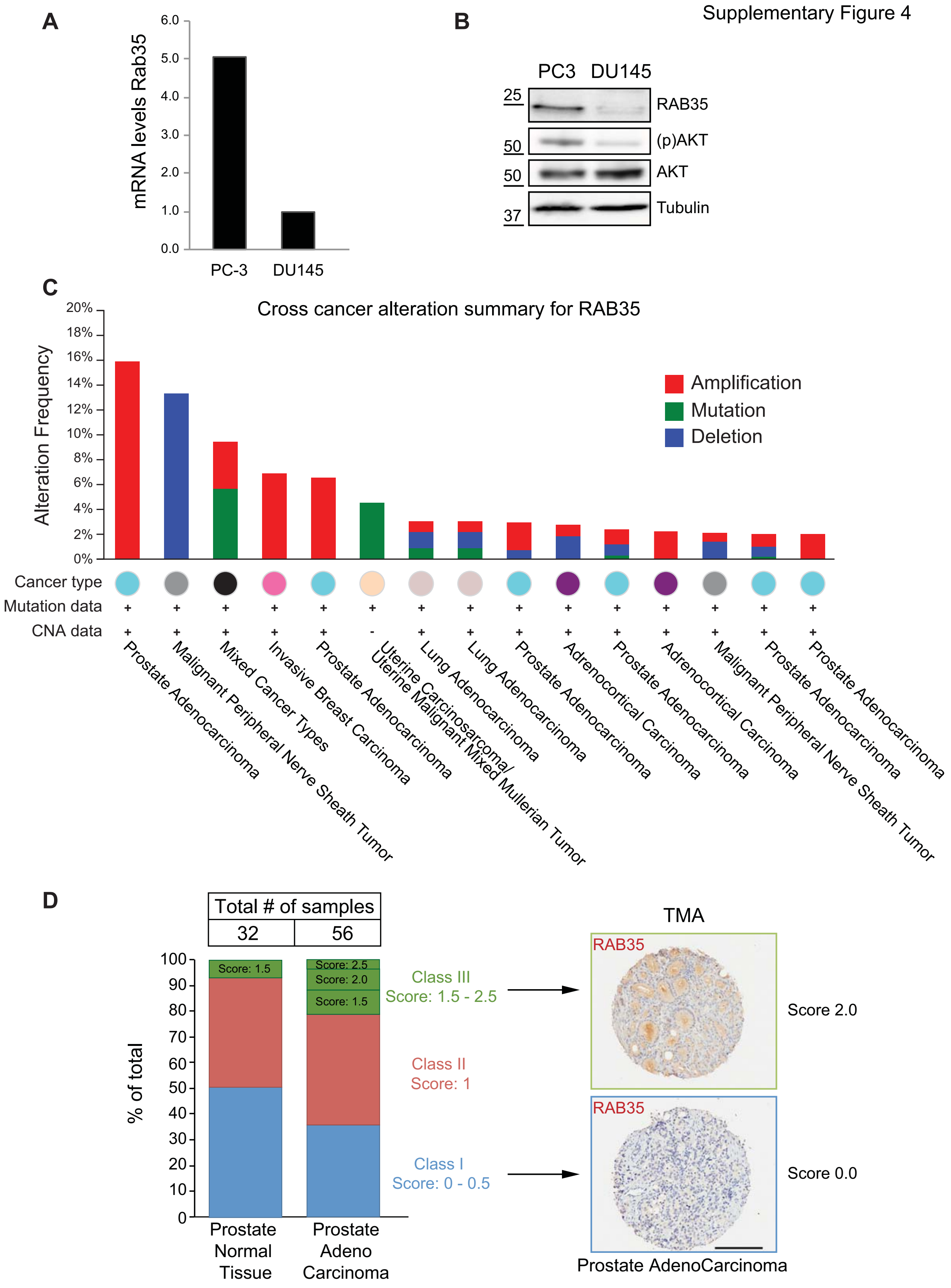

Supplementary Figure 4. RAB35 in cancer

(A) RAB35 mRNA level from PC3 and DU145 cells was measured by qRTPCR analysis.

(B) Total cell lysates from PC3 and DU145 cells were analysed by Western blot to detect total and phosphorylated levels of the indicated proteins. Tubulin was used as loading control.

(C) Summary of genomic alterations of RAB35 across different human tumor types and cancer cell lines retrieved from The Cancer Genome Atlas at cBioPortal (http://www.cbioportal.org/).

(D) RAB35 expression levels in normal and acinar adenocarcinoma prostate tissues. The TMA containing 56 prostate adenocarcinoma and 32 normal prostate tissues was used to analyse RAB35 expression by IHC. The specificity of the antibody was tested in paraffin-included control, RAB35-silenced or RAB35-expressing MEFs (not shown). *Left:* samples were scored according to the intensity of the staining of TMA and divided into 3 classes: class I, score = 0 – 0.5; class II, score = 1; class III, score 1.5 – 2.5. The percentage of tumors belonging to the 3 classes is reported in the graph. *Right:* representative images of class I and class III prostate adenocarcinoma. Scale-bar, 200μm.

Supplementary Figure 5-Uncropped gels#1

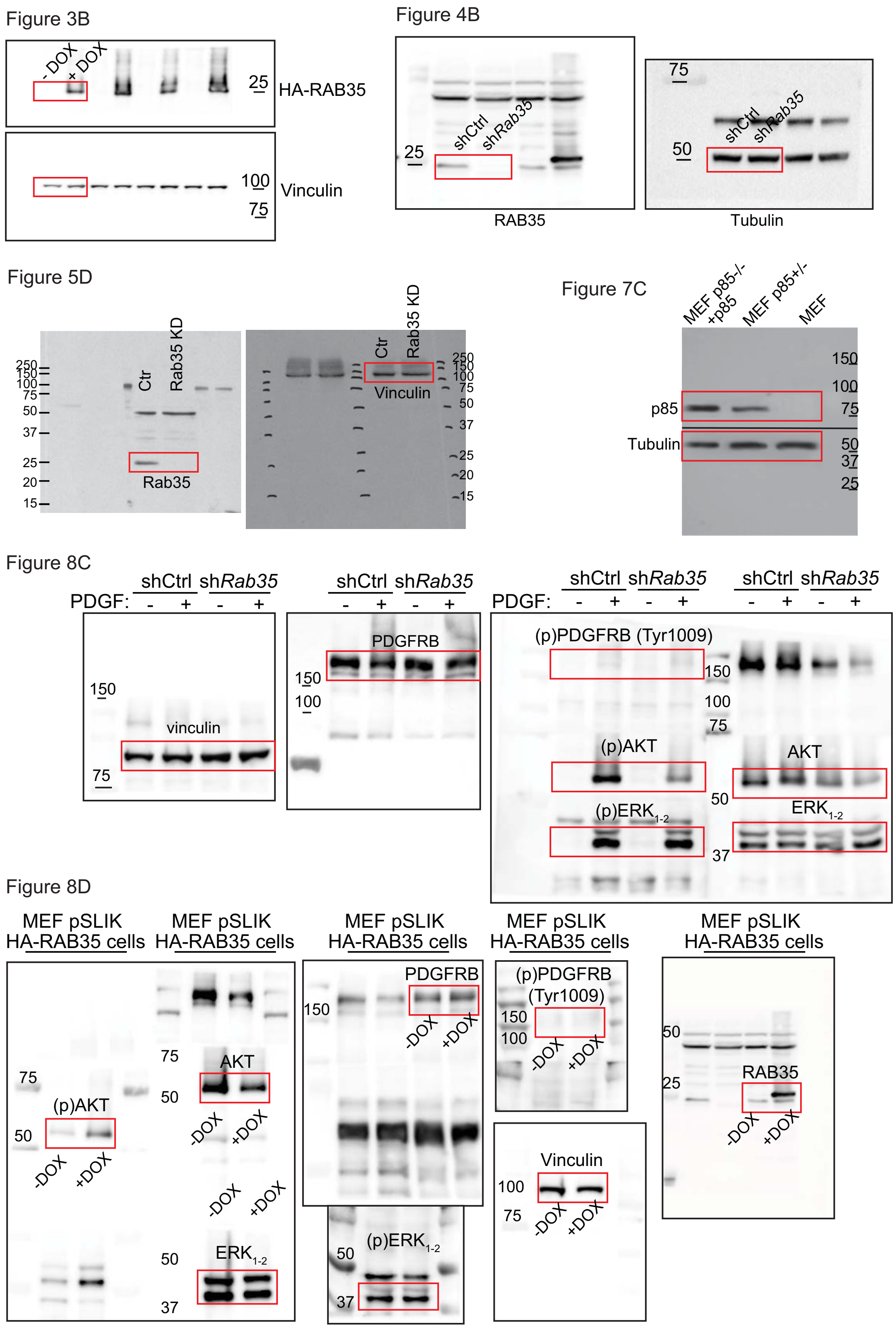

Supplementary Figure 5. Uncropped gels#1

Please note that gels from Fig 5D and 7C are auto-radiography films

Supplementary Figure 6-Uncropped gels#2

Figure 8E

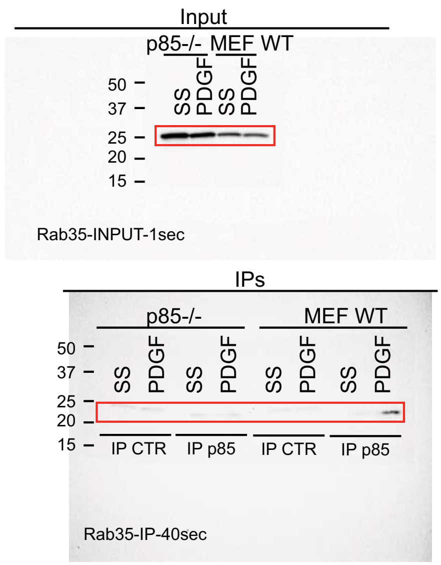

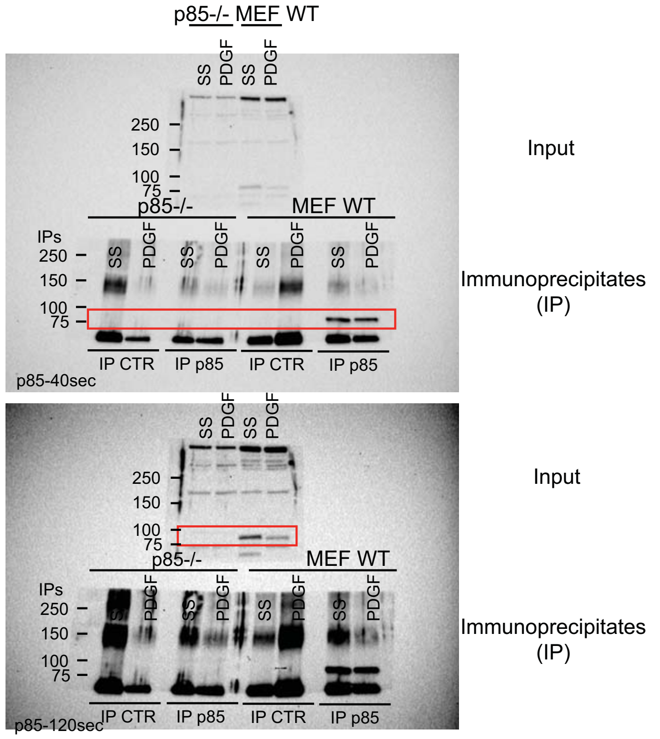

Figure 8F

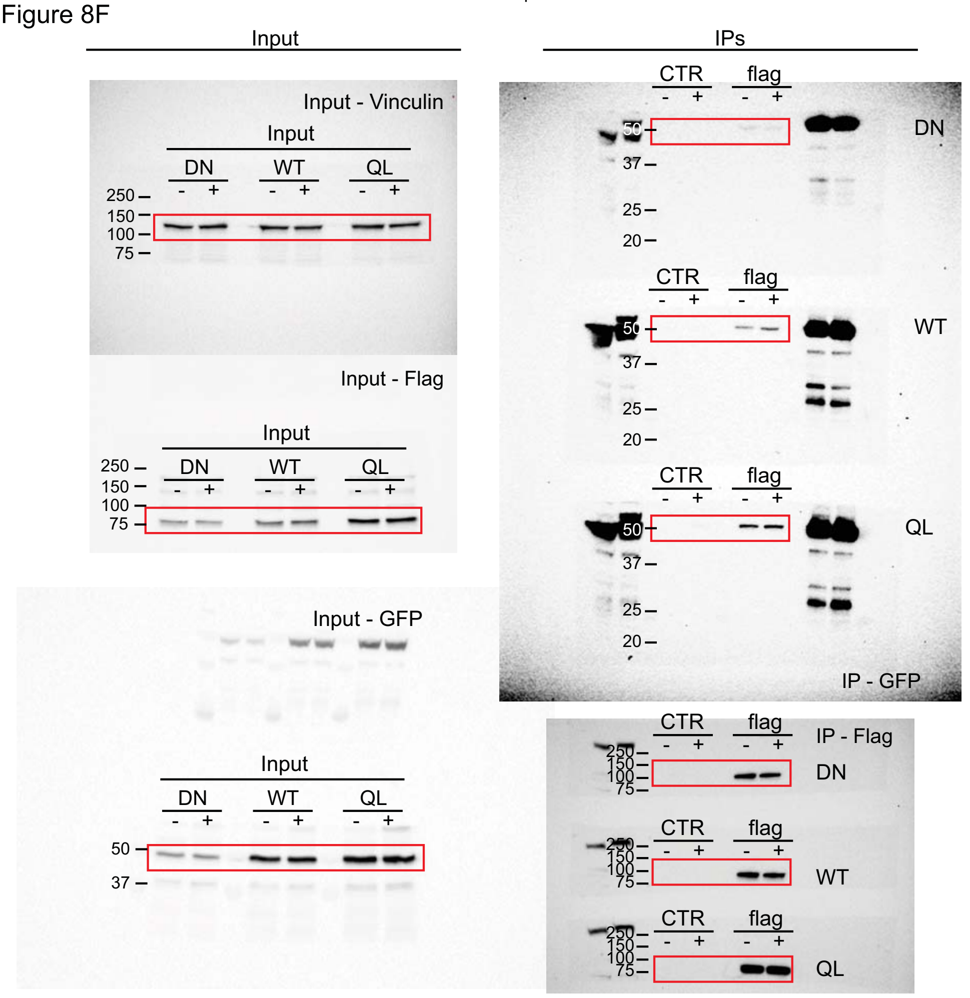

Supplementary Figure 6. Uncropped gels#2

Supplementary Figure 2A

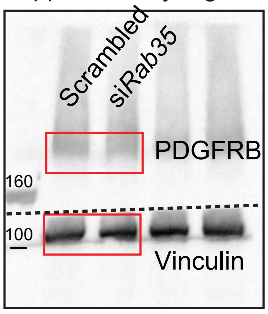

Supplementary Figure 2B

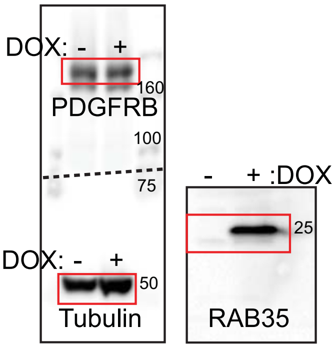

Supplementary Figure 3A

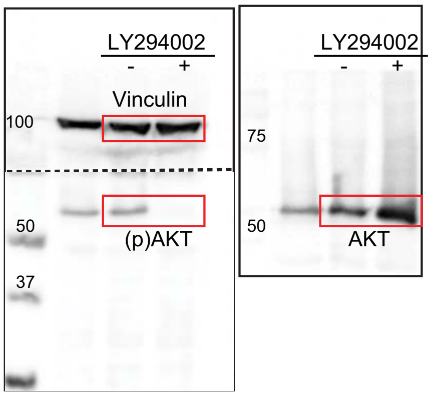

Supplementary Figure 3B

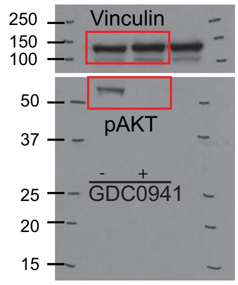

SupplementaryFigure 3C

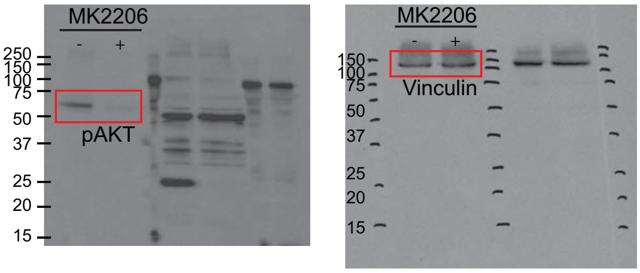

Supplementary Fig. 3E

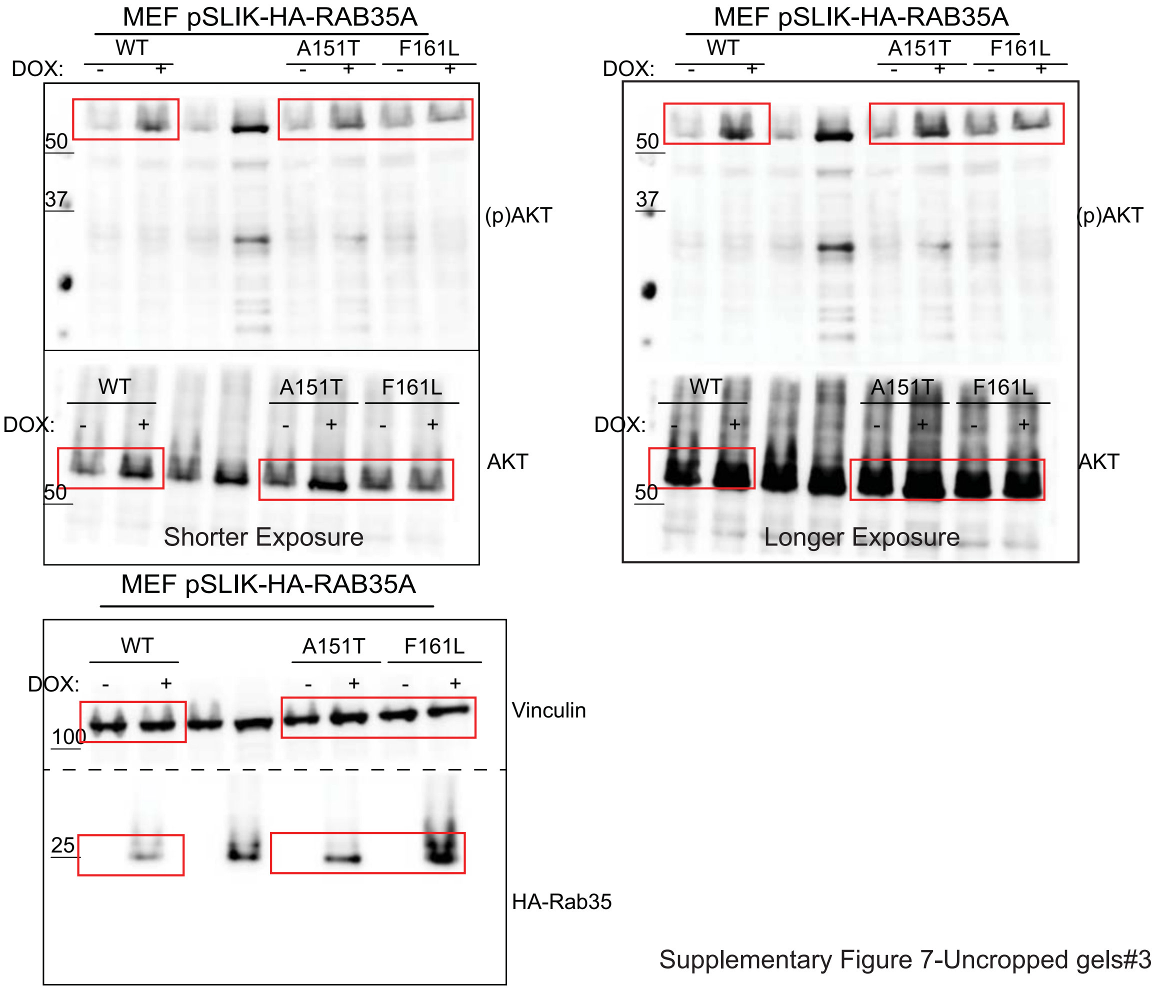

Supplementary Figure 7-Uncropped gels#3

Supplementary Figure 7. Uncropped gels#3

Supplementary Fig. 4B

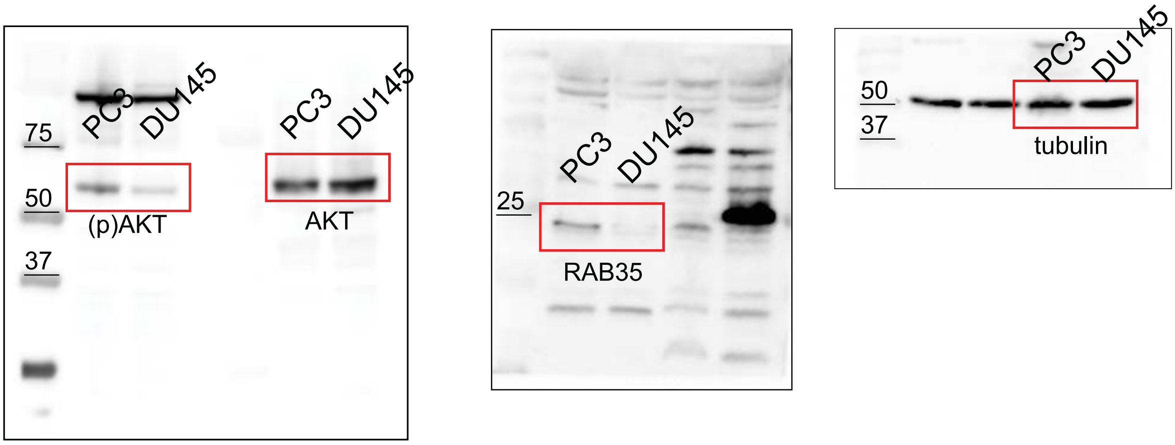

Supplementary Figure 8-Uncropped gels#4

Supplementary Figure 8. Uncropped gels#4
